# Artificial Neutrophils Against Vascular Graft Infection

**DOI:** 10.1002/advs.202402768

**Published:** 2024-06-14

**Authors:** Wentao Jiang, Huizi Xu, Zheng Gao, Ziyu Wu, Zichun Zhao, Jun Wang, Yawen Wu, Haifeng Ke, Chun Mao, Mimi Wan, Min Zhou

**Affiliations:** ^1^ Department of Vascular Surgery Cardiovascular center Nanjing Drum Tower Hospital The Affiliated Hospital of Nanjing University Medical School Nanjing 210008 China; ^2^ National and Local Joint Engineering Research Center of Biomedical Functional Materials School of Chemistry and Materials Science Nanjing Normal University Nanjing 210023 China; ^3^ Institute for Life and Health Nanjing Drum Tower Hospital Nanjing Normal University Nanjing 210023 China; ^4^ Department of Vascular Surgery Nanjing Drum Tower Hospital Clinical College of Nanjing University of Chinese Medicine Nanjing 210008 China

**Keywords:** artificial neutrophils, chemotaxis, staphylococcus aureus infections, vascular graft infection

## Abstract

Efficient neutrophil migration to infection sites plays a vital role in the body's defense against bacterial infections and natural immune responses. Neutrophils have a short lifespan and cannot be mass‐cultured in vitro. Therefore, developing more stable artificial neutrophils (AN) in a controllable manner has become a research focus. However, existing AN lack chemotaxis, which is the ability to migrate toward high‐signal‐concentration positions in a dynamic blood‐ flow environment. Supplying AN with chemotaxis is key to designing AN that are more similar to natural neutrophils in terms of morphology and function. In this study, micrometer‐sized, spherical, biocompatible AN are developed. These AN consist of zeolitic imidazolate framework‐8 nanoparticles encapsulating two enzymes, coacervate droplet frameworks, and outer phospholipid bilayers carrying enzymes. The AN exhibit responsiveness to elevated hydrogen peroxide levels at inflammation sites, actively chemotaxing toward these sites along concentration gradients. They also demonstrate effective combat against *Staphylococcus aureus* infections. The capabilities of the AN are further validated through in vitro experiments and in vivo evaluations using vascular graft infection models. This study replicates natural neutrophils in terms of chemical composition, functionality, and physiological impact. It introduces new ideas for advancing the development of advanced artificial cells.

## Graphical abstract

Supplying artificial neutrophils (AN) with chemotaxis is key to designing AN more similar to natural neutrophils. Here, micrometer‐sized biocompatible AN are developed that exhibit responsiveness to elevated hydrogen peroxide levels at inflammation sites, actively chemotaxing toward these sites along concentration gradients, and combat against *Staphylococcus aureus* infections. The capabilities of the AN are further validated using vascular graft infection models.

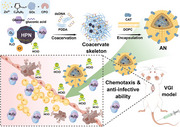

## 1 Introduction

Artificial cells (AC) are biomimetic systems crafted by researchers to explore the origins of life.^[^ ^1^ ^]^ By simulating natural cells across multiple dimensions such as morphology, growth, division, gene replication, and protein expression, researchers gain insights into the intricate biochemical interactions within and between cells.^[^ ^2^ ^]^ As AC construction technology progresses, some researchers are attempting to replicate specific cells in multiple dimensions, including their morphology, properties, and in vivo function. This endeavor poses a substantial challenge in AC design and is regarded as a pivotal step for the extensive application of AC in the biomedical field.^[^ ^3^ ^]^

Neutrophils are the most abundant white blood cells in the human blood.^[^ ^4^ ^]^ They can help resist invading pathogens by quickly migrating to the inflamed area, which is one of the crucial defense mechanisms in the body. Neutrophils play a vital role in clearing infections by destroying pathogens through various means, including damage caused by reactive oxygen species, primarily hypochlorous acid (HClO).^[^ ^4^, ^5^ ^]^ However, neutrophils have a short lifespan and typically survive in blood for less than 24 h. Moreover, they cannot be mass‐cultured in vitro because of their lack of proliferative ability.^[^ ^6^ ^]^ Consequently, researchers are trying to mimic neutrophils when designing AC, and developing more stable artificial neutrophils (AN) in a controllable manner has become a research focus.

Various methods for the preparation of AN have been studied. Antibiotics, being the most commonly used clinical anti‐infective strategy, were first introduced into AN. For instance, Lane et al. loaded Ceftazidime into block copolymer vesicles to construct AN with pH‐responsive drug release.^[^ ^7^ ^]^ Che et al. loaded Ciprofloxacin into vesicles assembled by macrophage membranes and artificial lipids to construct AN with lipopolysaccharide neutralization ability.^[^ ^8^ ^]^ Another class of methods for constructing AN is through functionalizing positively charged molecules, such as antimicrobial peptides and amines, onto the surface of polymer vesicles.^[^ ^9^ ^]^ Webster et al. prepared polymer vesicles by self‐assembly of PEG block poly(*d,l*‐lactide)[PEG‐b‐PDLLA], and modified the linear antimicrobial peptide PR‐39 on the surface of the vesicles to construct AN.^[^ ^10^ ^]^ Recently, there has been a focus on mimicking the production of ROS by natural neutrophils in the development of AN. Long et al. constructed a carbonaceous AN with an atomic catalytic center by carbonization, which can catalyze the generation of singlet oxygen and HClO.^[^ ^11^ ^]^ Zhou et al. loaded IR780 iodide onto the surface of gelatin microspheres to construct AN capable of generating singlet oxygen under laser irradiation at 808 nm.^[^ ^12^ ^]^ These studies have shown that AN can have some anti‐infective effects at the site of infection.

However, existing AN lack chemotaxis, which is the ability to migrate toward high‐signal‐concentration positions in a dynamic blood‐flow environment.^[^ ^13^ ^]^ Chemotaxis is a powerful response to inflammation in neutrophils and is crucial for their anti‐infective effects.^[^ ^14^ ^]^ The absence of chemotaxis prevents AN from actively aggregating and accumulating at the site of inflammation, considerably weakening their anti‐infective effects. Endowing AN with chemotaxis is a key step for researchers to design AN that are more similar to natural neutrophils in terms of morphology and function.

In this study, we engineered micrometer‐sized spherical biocompatible AN capable of active chemotaxis toward inflammation sites and possessing anti‐infective abilities based on HClO production for combating infections. To achieve this, we initially fabricated HClO‐producing nanoparticles (HPN) by encapsulating glucose oxidase (GOx) and chloroperoxidase (CPO) within a ZIF‐8 structure. This metal‐organic framework's spatial structure enhances the catalytic efficiency of GOx and CPO while improving their resilience to external stimuli such as pH and temperature.^[^ ^15^ ^]^ Subsequently, we constructed a micrometer‐sized coacervate AN framework that internally encapsulates HPN through liquid–liquid phase separation using double strand deoxyribonucleic acid (dsDNA) and an oppositely charged polymer.^[^ ^16^ ^]^ Finally, we obtained micrometer‐sized spherical AN by encapsulating the framework with a phospholipid bilayer carrying catalase (CAT).

**Figure 1:**
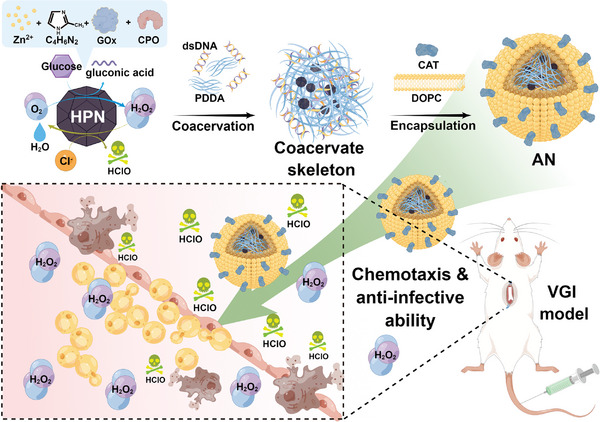
Schematic diagram of biomimetic construction, chemotaxis in the H_2_O_2_ concentration gradient, and anti‐infective capability of AN in the VGI mouse model. Created using Figdraw.

The outermost bilayer of the AN enhances biocompatibility and confers chemotaxis in response to the H_2_O_2_ concentration gradient.^[^ ^17^ ^]^ This implies that under physiological conditions, AN will react to elevated H_2_O_2_ levels at inflammation sites and actively migrate to these areas. Simultaneously, AN can catalyze glucose and Cl^−^ in the physiological milieu to generate HClO, thereby effectively exerting anti‐infective capabilities (**Figure** **1**).

To validate the aforementioned abilities of AN, we focused on vascular graft infection (VGI), a postoperative infectious disease associated with high mortality and disability rates in vivo. We successfully confirmed the chemotactic behavior and anti‐infective ability of AN through both in vivo and in vitro experiments.^[^ ^18^ ^]^ The successful incorporation of chemotaxis into AN marks a significant stride toward AC assuming certain natural cell functions. In essence, by mimicking the anti‐infective attributes of natural neutrophils, we endowed AN with chemotaxis and circumvented the short lifespan of natural neutrophils. These advantages, coupled with controllable batch preparation, offer innovative concepts and technical backing for the widespread application prospects of AC in the future.

## 2 Results

### 2.1 Synthesis and Characterization of AN

**Figure 2:**
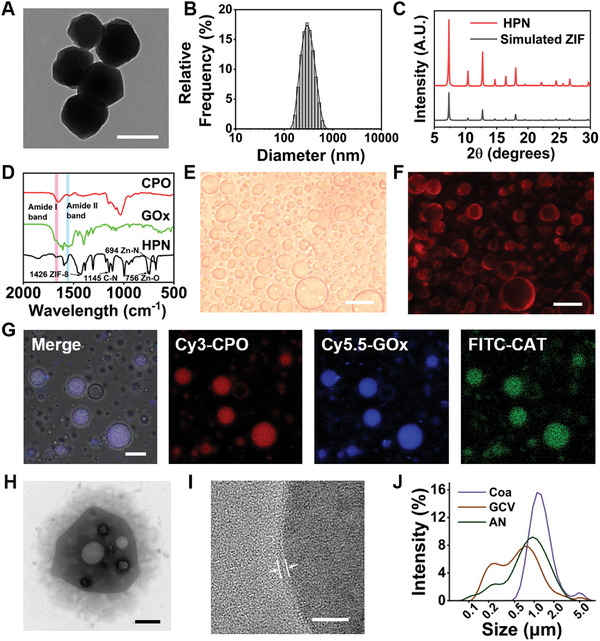
Assembly and characterization of AN. A) TEM image of HPN (scale bar, 200 nm). B) Hydrodynamic size of HPN determined via DLS measurement. C) PXRD patterns of HPN compared to the standard simulated ZIF. D) FT‐IR spectra depicting CPO, GOx, and HPN. E) Optical bright field image with F) corresponding red fluorescence image of DiI‐stained GCV microdroplets (scale bar, 10 µm). G) Confocal images illustrating Cy3‐CPO (red), Cy5.5‐GOx (blue), and FITC‐CAT (green) in AN (scale bar, 10 µm). H) TEM image of AN (scale bar, 500 nm). I) TEM image of the lipid bilayer surrounding AN (scale bar, 20 nm). J) DLS measurement of coacervate microdroplets without membrane, GCV, and AN.

As previously mentioned, zeolitic imidazolate framework‐8 (ZIF‐8) was selected as the metal‐organic framework (MOF) structure for encapsulating GOx and CPO. It was synthesized in an aqueous solution at 25 °C to preserve enzyme activity. The small pore size of ZIF‐8 helps prevent enzyme leakage and maintains the biocatalytic activity of GOx and CPO under physiological conditions.^[^ ^15^ ^]^ The resulting ZIF‐8 nanoparticles containing the enzymes are referred to as HPN. Transmission electron microscopy (TEM) images reveal that HPN exhibit a polyhedral crystalline structure with uniform size (**Figure** **2**A). The hydrodynamic diameter of HPN was determined to be ≈301 nm through dynamic light scattering (DLS) analysis (Figure 2B). Powder X‐ray diffraction (PXRD) confirmed the crystal structure of HPN, which was consistent with the simulated ZIF‐8 PXRD pattern (Figure 2C). Further characterization and verification of HPN's structure and the encapsulation of GOx and CPO were conducted using Fourier‐transform infrared spectroscopy (FT‐IR) spectra. As illustrated in Figure 2D, HPN exhibited characteristic peaks of ZIF‐8, including the ZIF‐8 ring at 1426 cm^−1^, C─N at 1145 cm^−1^, Zn─O at 756 cm^−1^, and Zn─N at 694 cm^−1^.^[^ ^19^ ^]^ The amide I and amide II bands of GOx and CPO proteins were observed ≈1650–1690 and 1540–1570 cm^−1^, respectively, indicating that these two enzyme proteins were successfully encapsulated in HPN.^[^ ^20^ ^]^

A membrane‐free coacervate microdroplet framework of AN was engineered using electrostatically mediated liquid‐liquid phase separation in a water mixture of poly (diallyldimethylammonium chloride) (PDDA) and dsDNA, creating AN with a spherical structure and phospholipid bilayer similar to that of natural neutrophils. During preparation, HPN dispersion was introduced into the dsDNA solution, yielding PDDA/HPN/dsDNA membrane‐free coacervate microdroplets successfully loaded with HPN (Figure S1, Supporting Information). These membrane‐free coacervate microdroplets were subsequently encapsulated with a continuous phospholipid membrane composed of 1,2‐dioleoyl‐sn‐glycero‐3‐phosphocholine (DOPC), forming stable giant coacervate vesicles (GCV) dispersion. The GCV appeared as discrete spherical droplets with a size ranging from 2 to 10 µm, as verified by bright‐field and fluorescence microscopy images. Upon staining with the lipophilic phospholipid bilayer probe DiI, these GCV exhibited a bright red fluorescent shell, indicating the successful encapsulation of DOPC (Figure 2E,F). Finally, CAT was loaded onto the surface phospholipid membrane of the GCV by combining it with palmitic acid (PA; hexadecanoic acid). The resulting CAT‐loaded GCV represented the target product, AN.

To confirm the simultaneous loading of all three enzymes (GOx, CPO, and CAT) onto AN, we labeled them with Cy5.5, Cy3, and fluorescein5(6)‐isothiocyanate (FITC), respectively, and employed confocal laser scanning microscopy (CLSM) to observe fluorescence colocalization. The results demonstrated overlapping fluorescence of all three enzymes, indicating their successful loading onto AN (Figure 2G). Morphology and structure characterization of AN were performed using TEM, revealing a dense interior formed by liquid–liquid phase separation and HPN encapsulation (Figure 2H). Additionally, irregularities in AN shape and a light‐colored bubble‐like spherical structure inside were noted, likely attributed to irregular sample shrinkage during the drying process.^[^ ^21^ ^]^ The enzyme‐loaded phospholipid membrane structure on AN's periphery was observed at higher magnification (Figure 2I).

Further characterization included assessing the hydrodynamic diameter distribution of spherical droplets at each preparation stage using DLS. The proportion of large‐sized spherical droplets gradually increased during the preparation of AN due to phospholipid membrane encapsulation, CAT loading, and droplet fusion effects (Figure 2J). The final AN diameter ranged from 2 to 10 µm, facilitating smooth movement in blood vessels and enabling its chemotactic function, considering human blood vessel thickness.^[^ ^22^ ^]^ To determine the surface charge positivity of AN, we conducted a measurement of the zeta potential of AN in water. As shown in Figure S2 (Supporting Information), the zeta potential for AN is −7.92 mV, indicating that the surface charge of AN is negative, same as that of native neutrophils.

### 2.2 Chemotaxis, HClO‐Producing Ability, and Stability of AN

**Figure 3:**
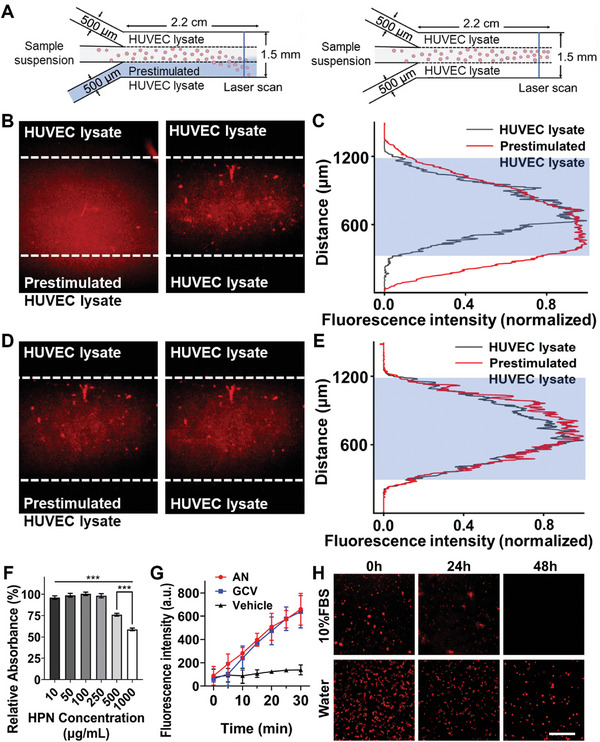
Chemotaxis, HClO‐producing ability, and stability of AN. A) Schematic illustration of the three‐inlet, one‐outlet microfluidic device. B) Representative CLSM images depicting microfluidic channels for AN or D) GCV traversing the middle channel, with normal/prestimulated HUVEC lysates passing through the channels on each side (n = 3 independent experiments). C,E) Graphs displaying fluorescence intensity distribution normalized to a value between 0–1. A typical volume flow rate of 0.4 mL h^−1^ through each inlet was maintained to ensure interaction time. F) Quantitative analysis of AO7 decolorization. G) Fluorescence changes observed in AN, GCV, and Vehicle in PBS solutions with glucose during a 30‐min incubation. H) CLSM fluorescence images of Cy5.5‐labeled AN in 10% FBS or water at different time points (scale bar, 40 µm). Statistical significance was determined using one‐way ANOVA with a Tukey post hoc test (^***^ *p* <0.001). n = 3 independent experiments, and mean values and standard deviations (SD) are presented.

Natural neutrophils often react to inflammatory factors in the bloodstream by initiating chemotaxis, migrating toward the disease sites.^[^ ^23^ ^]^ To assess the chemotaxis of AN in response to elevated H_2_O_2_ levels at inflammation sites, we monitored the directional movement of AN in a three‐in‐one microfluidic channel (refer to “Experimental Section” for details).^[^ ^24^ ^]^ As shown in **Figure** **3**A, we created an H_2_O_2_ concentration gradient by pumping lipopolysaccharide (LPS)‐prestimulated inflammatory human umbilical vein endothelial cell (HUVEC) lysate into the lower channel and normal HUVEC lysate into the upper channel as a control. A control group with normal HUVEC lysate in both channels mimicked regular blood flow with lower H_2_O_2_ concentration. The volume flow rate of the channel was set at 0.4 mL h^−1^ to simulate capillary blood flow rates in vivo (0.3–0.7 mm s^−1^). We performed optical scanning (30 fps) near the outlet using CLSM to assess the chemotactic displacement of AN by monitoring changes in fluorescence intensity perpendicular to the flow direction. In Figure 3B, the Cy5.5‐labeled AN fluid's fluorescence signal is observed moving downward toward higher H_2_O_2_ concentration, indicating migration of AN along the H_2_O_2_ concentration gradient in the fluid environment. Quantification revealed the strongest fluorescence intensity at the middle position, gradually decreasing on both sides, with the fluorescence signal spreading from the middle to the lower channel with high H_2_O_2_ concentration. Conversely, Figure 3C shows that AN with normal HUVEC lysate in both channels did not exhibit lateral fluorescence spread, serving as a control. We also used Cy5.5‐labeled GCV without CAT as a control to assess the impact of horizontal flow due to fluid inflow into the channel. Figure 3D indicates that GCV did not exhibit notable chemotactic behavior regardless of the H_2_O_2_ concentration gradient, confirmed by the fluorescence signal not spreading to one side (Figure 3E). The dynamic video Movie S1 (Supporting Information) of the microfluidic channel corroborated these findings. These results demonstrate that AN can sense H_2_O_2_ concentration gradients in dynamic fluid environments, moving toward higher H_2_O_2_ concentrations, underscoring the designed AN's ability to respond to inflammatory chemical signals near inflammation sites and achieve chemotaxis. We then conducted further experiments to investigate how AN respond to different concentrations of H_2_O_2_. To achieve this, we have recorded the chemotaxis data of AN in a three‐in‐one microfluidic channel under various H_2_O_2_ concentration gradients. Our findings show that AN demonstrate a more prominent shift toward the H_2_O_2_ side under higher H_2_O_2_ concentration gradients (Figure S3, Supporting Information). This suggests that AN exhibit a stronger chemotaxis response toward higher H_2_O_2_ concentration gradients.

We investigated whether AN can produce HClO to combat pathogens, akin to natural neutrophils. Initially, we set up different groups to explore the optimal ratio of GOx/CPO for the maximum production efficiency of HClO and to avoid raw material waste. These groups included 0.5 mg GOx/45 U CPO, 1 mg GOx/45 U CPO, 2 mg GOx/45 U CPO, 3 mg GOx/45 U CPO, and 4 mg GOx/45 U CPO. The research showed that when the mass of GOx was increased, the HClO production efficiency reached its highest point when the ratio was 2 mg GOx/45 U CPO, as shown in Figure S4 (Supporting Information). The efficiency of HClO production did not significantly improve with a higher proportion of GOx mass. Therefore, we chose this enzyme ratio for the subsequent synthesis of AN. Then, we employed orange II sodium salt (AO7), an azo dye, to examine the bleaching efficiency of HClO generated by HPN.^[^ ^25^ ^]^ The results revealed that as the HPN concentration increased, the color of AO7 progressively degraded and faded (Figure S5, Supporting Information). Utilizing an enzyme‐linked immunosorbent assay (ELISA), we measured the relative absorbance at 484 nm, further confirming HPN's capacity to generate HClO and decolorize AO7 (Figure 3F).

Subsequently, we employed an HClO fluorescence probe to evaluate the HClO release efficiency of AN within a physiological environment. Our findings demonstrated that both AN and GCV containing HPN exhibited an increase in fluorescence intensity over the 30 min incubation period (Figure 3G). This indicated that AN and GCV could steadily and gradually release HClO over time. In contrast, the Vehicle group, lacking HPN, maintained consistently low fluorescence intensity, signifying its inability to release HClO. In our study, we used a high‐glucose Dulbecco's modified Eagle medium (DMEM) that contained substrates required for AN to catalyze HClO generation, such as glucose, O_2_, and Cl^−^. To make sure that AN could actually generate HClO in culture medium conditions, we conducted an experiment using an HClO fluorescent probe to observe the fluorescence changes in AN in culture medium conditions during a 30 min incubation. The results, shown in Figure S6 (Supporting Information), indicate that AN can produce HClO in DMEM high‐glucose medium, which aligns with our expectations.

The typical lifespan of neutrophils in circulation is short, lasting less than 24 h. Extending their lifespan could potentially mitigate inflammation.^[^ ^5^, ^26^ ^]^ Given that AN are a bottom‐up synthesized biomimetic system mimicking natural neutrophils, their stability in a physiological environment is crucial. Insufficient stability might cause AN to lose structural integrity, diminishing its chemotactic and HClO release capabilities. Conversely, excessive stability could result in AN accumulation and tissue blockage. To evaluate integrity of AN, we labeled AN with Cy5.5 (8 × 10^5^ cells mL^−1^) and mixed them with a 10% fetal bovine serum (FBS) solution. We observed AN under CLSM at different time points (0, 24, and 48 h) and compared them with AN of the same density in water, serving as a control. The results showed a gradual decrease in red fluorescence and particle density in the 10% FBS group over 48 h, with complete degradation observed at 48 h. Conversely, AN exhibited better stability in water, exhibiting only partial degradation at 48 h (Figure 3H). We conducted an experiment to determine whether phospholipid bilayers and ZIF‐8 nanoparticles would degrade in a 10% FBS solution within 48 h. We took TEM images of AN after it had been standing in a 10% FBS solution for 48 h, and we measured DLS data for this solution. As shown in Figure S7A (Supporting Information), we observed no phospholipid bilayers or typical ZIF‐8 nanoparticles in the field of view, suggesting that AN had completely degraded after 48 h in the 10% FBS solution, including the phospholipid bilayers and ZIF‐8 nanoparticles. The DLS measurement graph of AN in a 10% FBS solution after 48 h also confirmed the degradation of ZIF‐8 nanoparticles and phospholipid bilayers (Figure S7B, Supporting Information). These findings indicate that AN can maintain good stability and a longer lifespan in water compared to natural neutrophils, highlighting their potential for storage and use in physiological environments.

### 2.3 In Vitro Biocompatibility and Antibacterial Ability of AN

**Figure 4:**
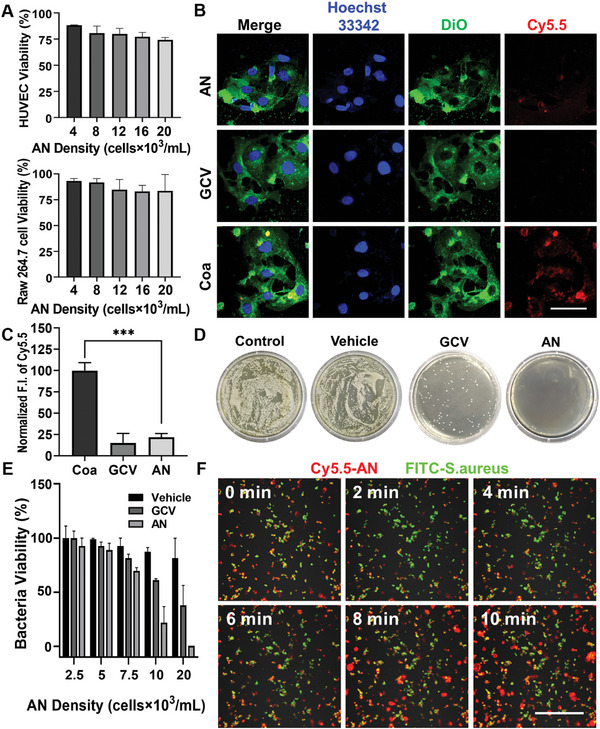
Biocompatibility and in vitro antibacterial effects of AN. A) Cell viability of HUVECs and Raw 264.7 cells treated with different concentrations of AN, assessed using the CCK‐8 assay. B) CLSM images depicting the colocalization of HUVECs with various materials after a 24‐h incubation period (scale bar, 50 µm). C) Quantitative analysis of Cy5.5‐AN fluorescence intensity. D) Representative images of *S. aureus* colonies on LB agar plates following co‐incubation with different materials. E) Relative bacterial viability of *S. aureus* post‐co‐incubation with different materials. F) CLSM images showing the colocalization of *S. aureus* (green) with AN (red) over a 10 min period (scale bar, 50 µm). Statistical significance was determined using one‐way ANOVA with a Tukey post hoc test (^***^ *p* <0.001). n = 3 independent experiments, and mean values and standard deviations (SD) are presented.

Endothelial cells and macrophages are key cell groups in blood vessels near inflammation sites, making it essential to evaluate the biocompatibility of AN with these cells. AN at varying densities (4–20 × 10^3^ cells mL^−1^) were incubated with HUVECs or murine macrophages Raw 264.7 cells for 24 h, and cell viability was measured using the CCK‐8 method. **Figure** **4**A illustrates that the viability of both cell types slightly decreased with increasing AN density but remained above 70%, indicating good biocompatibility.

AN, as artificial cells simulating natural neutrophils in HClO production, need to maintain their anti‐infective effects outside natural cells. Excessive uptake by HUVECs and other cells can significantly diminish their chemotaxis and anti‐infective ability, even negatively impacting the cells taking up AN.^[^ ^27^ ^]^ Therefore, we investigated the impact of AN's outer phospholipid membrane encapsulation on cell uptake. Cy5.5‐labeled AN (2 × 10^4^ cells mL^−1^) with phospholipid bilayer and CAT loading, GCV with phospholipid bilayer but without CAT loading, and membrane‐free coacervate microdroplets (Coa) lacking a phospholipid bilayer were incubated with HUVECs for 24 h and observed via CLSM. Results indicated that the fluorescence intensity of AN and GCV internalized by HUVECs was significantly lower than that of Coa (Figure 4B). Specifically, compared to Coa, the fluorescence intensity of GCV and AN taken up by HUVECs reduced by 24.2% and 12.6%, respectively (Figure 4C). This suggests that encapsulating the phospholipid bilayer effectively reduces AN uptake by vascular cells.

In this study, we examined the anti‐infective ability of AN as a mimic of natural neutrophils using two methods: the spread plate method and turbidity assay.^[^ ^28^ ^]^ *Staphylococcus aureus* (*S. aureus*), commonly found in VGI cases, was employed to evaluate AN, GCV, and Vehicle,^[^ ^29^ ^]^ where Vehicle denotes AN lacking HPN. Figure 4D reveals that both GCV and AN exhibited significantly fewer colonies compared to the blank control and Vehicle groups, indicating robust antibacterial activity due to their HClO generation ability. Various densities (2.5–20 × 10^3^ cells mL^−1^) of these materials were incubated with *S. aureus* for 8 h, and optical density at 600 nm (OD 600) was measured in the culture medium using the turbidity assay method. The antibacterial effects of GCV and AN positively correlated with density within the biocompatible range, peaking at 2 × 10^4^ cells mL^−1^ (Figure 4E). Among them, AN demonstrated the most potent inhibitory efficiency against *S. aureus*.

Additionally, we investigated the interaction between AN and *S. aureus* through co‐incubation observed using CLSM. Figure 4F and dynamic video Movie S2 (Supporting Information) depict that upon adding diluted AN dispersion to a phosphate buffered saline (PBS) suspension of *S. aureus*, AN progressively aggregated at the bacterial site within 10 min. As AN density increased in the observed field, the colocalization of bacteria and AN also increased, indicating the ability of AN to adhere to and capture *S. aureus*.

### 2.4 In Vivo Chemotaxis and Anti‐Infective Ability of AN

**Figure 5:**
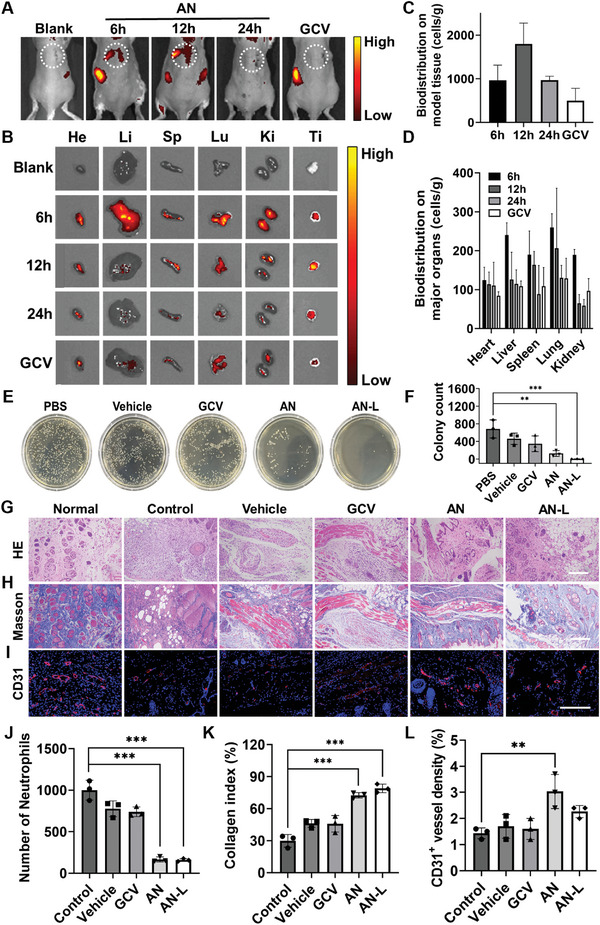
Assessment of chemotaxis and anti‐infective ability of AN. A) In vivo fluorescence images of VGI mouse models after tail vein injection of AN and GCV at different time intervals. The white circle indicates the modeled area. B) Ex vivo fluorescence images of tissues and major organs in the modeled area after tail vein injection of AN and GCV at different intervals, and C,D) corresponding quantitative statistical analysis. E) Representative image of *S. aureus* colonies on LB agar plates of VGI mouse models treated with different materials on day 8, with F) corresponding statistical analysis. G) HE, H) Masson, and I) CD31 staining images of the epidermal histological sections in different groups at day 8 (scale bar, 200 µm). Quantitative analysis includes, J) number of neutrophils, K) collagen index, and L) density of blood vessels. n = 3 independent experiments, and data are presented as mean ± SD. Statistical significance was determined using one‐way ANOVA with a Tukey post hoc test (^**^ *p* <0.01, ^***^ *p* <0.001).

Next, we conducted a study to evaluate AN's chemotaxis using an in vivo VGI model. The VGI mouse model was established based on the standard animal model utilized in previous VGI research. This involved subcutaneously implanting an artificial vascular graft into BALB/c mice and subsequently injecting a suspension of S. aureus.^[^ ^30^ ^]^ After 24 h, we administered Cy5.5‐labeled AN and GCV dispersions into the mice's tail veins and conducted in vivo fluorescence imaging at various time points. Our findings revealed a pronounced chemotactic effect of AN, with concentrated fluorescence observed at the site of modeling on the mouse's back (**Figure** **5**A). Notably, AN exhibited a peak fluorescence intensity at 12 h, which was significantly higher than that of GCV at the same time, indicating rapid accumulation of AN in infected tissues within a 12 h timeframe.

Further ex vivo fluorescence imaging and quantitative analysis of the modeling tissue corroborated these observations (Figure 5B,C). Additionally, we studied the biological distribution of AN in major organs at different time points post‐injection. It was observed that fluorescence intensity was notably elevated in all major organs at 6 h, gradually diminishing thereafter, and significantly decreasing after 24 h (Figure 5B). Quantitative analysis further revealed that AN accumulation per gram of tissue was highest in the liver and lungs at 6 h, followed by the spleen and kidneys, albeit at lower yet significant levels (Figure 5D). This suggests that AN can accumulate and undergo metabolic degradation in various organs. Importantly, AN did not induce organ damage or elevate inflammation levels in vivo. This was confirmed through hematoxylin‐eosin (HE) staining of major organs and blood routine examination, demonstrating AN's favorable biocompatibility in vivo (Figure S8, Table S1, Supporting Information).

We evaluated the therapeutic efficacy of AN, GCV, and Vehicle in treating VGI model mice via tail vein injection, alongside a control group treated with PBS. Additionally, we included an AN local injection group (AN‐L) to investigate AN's anti‐infective effects at the modeling site. After 8 days, we excised the inflamed tissue from the mice to quantify bacterial colonies. The results indicated that the AN‐L group exhibited the lowest number of *S. aureus* colonies on the agar medium, followed by the AN group with chemotactic ability (Figure 5E,F). Conversely, the GCV and Vehicle groups showed higher colony counts than the AN and AN‐L groups, indicating significant anti‐infective efficacy in both AN and AN‐L groups, with the AN‐L group demonstrating the most effective anti‐infective efficiency. Subsequently, we conducted HE, Masson, and CD31 staining to evaluate the modeling tissue. Compared with healthy tissue, infected tissue displayed numerous necrotic polymorphonuclear leukocytes and neutrophils.^[^ ^31^ ^]^ However, the AN‐L and AN groups exhibited a significant decrease in neutrophil numbers compared to the control group, surpassing the Vehicle and GCV groups (Figure 5G,J). Additionally, we observed a considerable area occupied by collagen fibers in tissue slices from the AN‐L and AN groups, with the fibers exhibiting an even and uniform arrangement, indicative of positive collagen regeneration effects (Figure 5H,K). Furthermore, CD31 staining revealed regenerated endothelial cells in the AN‐L and AN groups, indicating the formation of newly generated blood vessels following blood flow reconstruction in the modeling area. The number of newly formed blood vessels was significantly higher in the AN group compared to the control, GCV, and Vehicle treatment groups (Figure 5I,L). These in vivo anti‐infective results collectively suggest that the synthesized AN effectively mimic natural neutrophils, showcasing chemotaxis to infection sites and exerting anti‐infective effects.

We then added a rat abdominal aortic transplantation VGI model to verify the vascular application of our AN. Based on this model, we established three groups: AN tail‐vein‐injection group (AN group), AN in situ administration group (AN‐L group), and a control group (PBS group). Figure S9 (Supporting Information) shows our successful establishment of a rat abdominal aortic transplantation model. An expanded polytetrafluoroethylene vascular graft patch (1.5 mm^2^) was sutured at the anterior wall of the rat abdominal aorta. 100 µL of *S. aureus* (2 × 10^7^ CFU mL^–1^) was inoculated onto the graft surface. In the control group, we observed significant infection and abscess accumulation around the vascular grafts, while the infection was significantly reduced in both the tail vein injection and in situ administration groups (Figure S10A, Supporting Information). Additionally, in the bacterial spread plate method of vascular grafts, the colony count in both the tail vein injection and in situ administration groups was significantly lower than that in the control group (Figure S10B,C, Supporting Information). The conclusions of these experiments are consistent with those of the subcutaneous transplantation model experiments, demonstrating the excellent anti‐infective ability of AN in VGI in both animal models.

## 3 Conclusion

In conclusion, we have successfully developed AN capable of mimicking natural neutrophils. AN exhibit a micrometer‐sized spherical structure and demonstrate active chemotaxis toward inflammatory sites, producing HClO to combat infections. Our experiments confirm the successful construction of AN, showcasing their sustained HClO production under physiological conditions. Microfluidic studies illustrate the chemotactic behavior of AN in response to H_2_O_2_ concentration gradients in dynamic fluids, with the phospholipid bilayer reducing cellular uptake effectively. In vitro assessments validate AN's biocompatibility and efficacy against *S. aureus* infections. Animal models utilizing the VGI model further demonstrate chemotaxis and accumulation of AN at the infection site, along with their anti‐infective and tissue repair capabilities due to HClO release.

Moreover, mature human neutrophils cannot be cultured in large quantities in vitro. This is a major obstacle to research into the treatment of diseases based on neutrophils and their engineered products. The AN designed in this study can be stored in water with good stability, which is convenient for scalable production and timely dosing at different time points. In addition, neutrophils generally circulate in the body for no more than 24 h. AN exhibit a longer lifespan in physiological environments compared to natural neutrophils. The characteristics possessed by AN make them exhibit unique advantages in disease treatment applications, including VGI.

This study marks a significant advancement in AN development. AN that produced ROS were found to be a better solution to bacterial resistance than previously‐studied AN loaded with antibiotic. Additionally, they were less toxic to mammalian cells than AN loaded with antimicrobial peptides and amines. Our study involved the introduction of chemotaxis, which made AN function more like native neutrophils. It is significant for the field of AC, as researchers are trying to simulate specific natural cells in terms of their structure, properties, and in vivo functions. The successful construction of AN in our study demonstrates the potential of AC in the field of disease treatment.

There is still a lot of room for improvement in AN. For example, unlike neutrophils in the circulation, AN cannot detect the level of signal concentration from a remote injection site to the site of infection. This is because when H_2_O_2_ is produced at the site of inflammation, it quickly gets diluted once it enters the bloodstream. It is only near infection site that AN can sense the concentration gradient of H_2_O_2_ and move toward areas with higher signal concentrations through chemotaxis in the blood vessels. In addition, AN are unable to achieve the controllable release of HClO. In future research, researchers can combine synthetic materials with natural materials such as cell membranes and DNA to construct AN that have a partial neutrophil metabolism mechanism. Such AN will perform better in sensing circulating inflammatory cytokines, like IL‐1β. It will be a milestone in the development of AN. Additionally, if AN can crawl on the blood vessel wall and control the release of ROS, it will become more similar to neutrophils. Therefore, these improvements to AN will bring it closer to native neutrophils and further promote its application and clinical translation in disease treatment.

## 4 Experimental Section

### Materials

Zinc nitrate hexahydrate (Zn (NO_3_)_2_·6H_2_O) was procured from Sinopharm Chemical Reagent Co., Ltd. (Shanghai, China). CPO and dsDNA were obtained from Sigma–Aldrich Co., Ltd. (Darmstadt, Germany). 2‐methylimidazole, GOx, PDDA, FITC, AO7 were purchased from Aladdin Chemistry Co., Ltd. (Shanghai, China). Cy5.5‐NHS ester (non‐sulfonated) was purchased from APExBIO Technology LLC. (Houston, USA). Cy3‐NHS (non‐sulfonated) was purchased from Duofluor Inc. (Wuhan, China). Agar was purchased from Beyotime Biotechnology Inc. (Shanghai, China). Palmitic acid N‐hydroxysuccinimide ester (PA‐NHS) and DiI were acquired from Shanghai Yuanye Biotechnology Co., Ltd. (Shanghai, China). CAT and DOPC were obtained from Shanghai Macklin Biochemical Co., Ltd. (Shanghai, China). The fluorimetric hypochlorite assay kit was purchased from AAT Bioquest, Inc. (California, USA). Penicillin‐Streptomycin was purchased from LONSERA, Suzhou Shuangru Biotechnology Co., Ltd. (Suzhou, China). 0.25% Trypsin, PBS (1×, pH 7.2), and serum‐free cell freezing medium were purchased from KeyGEN BioTECH Co., Ltd. (Nanjing, China). Pipettes were sourced from DLAB Scientific Co., Ltd.

### Preparation and Characterization of HPN

The synthesis of HPN followed established protocols as described in the literature.^[^ ^32^ ^]^ Briefly, a mixture containing Zn (NO_3_)_2_·6H_2_O (40 mg), CPO (45 U), GOx (2 mg), and 2‐methylimidazole (0.77 g) was dissolved in 5 mL of deionized (DI) water. The resulting mixture was stirred at room temperature for 5 min, leading to the formation of slightly yellow nanocrystals. These nanocrystals were then obtained by centrifugation and washed several times with DI water. The final product was obtained through freeze drying. The morphology of HPN was examined using transmission electron microscopy (JEM‐2100, Hitachi, Japan), and the size distribution was measured through DLS (Zetasizer Nano Z, Malvern, UK). The structural characterization of HPN was carried out using XRD (D/max 2500/PC, Rigaku, Japan), while the encapsulation of CPO and GOx was confirmed using FT‐IR (Nexus‐670, Nicolet, USA).

Furthermore, the preparation of HPN‐Cy5.5 involved dissolving 2 mg of GOx in 4 mL of water, followed by the addition of 200 µL of Cy5.5‐NHS solution (1 mg mL^−1^ in dimethyl sulfoxide[DMSO]) at 37 °C and stirring for 12 h in the dark. CPO (45U) and Zn (NO_3_)_2_·6H_2_O (40 mg) were then added to the solution to achieve a final volume of 5 mL. After stirring at 25 °C for 5 min, nanocrystals were obtained by centrifugation, washed with DI water several times, and finally freeze‐dried to obtain the product.

The preparation of HPN‐Cy5.5‐Cy3 followed a similar procedure. Initially, 2 mg of GOx was dissolved in 2 mL of water, and then 200 µL of Cy5.5‐NHS solution (1 mg mL^−1^ in DMSO) was added and stirred at 37 °C for 12 h in the dark. Concurrently, 45 U of CPO and 200 µL of Cy3‐NHS solution (1 mg mL^−1^ in DMSO) were mixed in 2 mL of water at 37 °C and stirred for 12 h in the dark. Subsequently, these two solutions were combined with 40 mg of Zn (NO_3_)_2_·6H_2_O to reach a final volume of 5 mL. The resulting mixture was stirred at room temperature for 5 min, followed by centrifugation, washing with DI water, and freeze‐drying to obtain the final product.

### Hydrophobic Modification of CAT

The hydrophobic modification of CAT with palmitic acid ester was carried out as follows.^[^ ^33^ ^]^ First, 0.4 mL of PA‐NHS solution in dry dimethyl sulfoxide (added in four portions of 0.1 mL every 2 h) was introduced into 6 mL of CAT solution (4 mg mL^−1^) in 1x PBS. The molar ratio of enzymes to the ester in the reaction mixture was maintained at 1:40. The reaction mixture was incubated for 8 h in a thermostat set at 30 °C while stirring. Subsequently, the mixture underwent dialysis using PBS for three cycles. Following dialysis, the solution containing the hydrophobized CAT (PA‐CAT) was filtered through a 0.2 µm filter, then lyophilized, and stored in powder form.

For FITC labeling of the PA‐CAT, the process was integrated into the PA‐NHS conjugation step. Specifically, 1 mg mL^−1^ of FITC in dimethyl sulfoxide was combined with PA‐NHS and added to the CAT solution, resulting in a final concentration of 100 µg FITC per 1 mg CAT. The sample tube was shielded from light using foil and allowed to mix at room temperature for 2 h. Any unreacted FITC was eliminated through dialysis. The FITC‐labeled PA‐CAT was collected, stored in PBS buffer at −20 °C, and reserved for further use.

### Preparation and Characterization of AN

Membrane‐less PDDA/HPN/dsDNA coacervate microdroplet suspensions were initially prepared by combining 0.5 mL of dsDNA aqueous solution (low molecular weight, from salmon sperm, 10 mg mL^−1^), 0.3 mL of PDDA aqueous solution (average molecular weight 200–350 kDa, 10 mg mL^−1^), and 0.1 mL of HPN dispersion (10 mg mL^−1^).

For the preparation of GCV, 0.9 mL of a suspension of PDDA/HPN/dsDNA coacervate microdroplets was added to 45 µL of 20 mg mL^−1^ DOPC ethanol solution, resulting in a final weight ratio of 10% (DOPC: coacervate, w/w). Following aging for over 2 h at room temperature, a suspension of membraned coacervate vesicles was obtained. After staining with DiI (10 µm) for 20 min, brightfield and fluorescence microscopy images of GCV were captured using 100x microscope objectives. Additionally, GCV‐Cy5.5 were prepared by substituting HPN with HPN‐Cy5.5.

AN were prepared as follows. A 1 mL suspension of GCV (10 mg mL^−1^) was mixed with 50 µL of PA‐CAT solution (5 mg mL^−1^). After incubation for 120 min and subsequent centrifugation for 5 min at 20  ×  g, AN were obtained. The number density of AN in suspension was determined using a flow cytometer (Accuri C6, BD Biosciences, USA). The appearance of AN was observed using a transmission electron microscope (JEM‐2100, Hitachi, Japan). The size distributions of PDDA/HPN/dsDNA coacervate microdroplets, GCV, and AN were measured through DLS (Zetasizer Nano Z, Malvern, UK). Furthermore, AN‐Cy5.5 was prepared by replacing HPN with HPN‐Cy5.5. AN‐Cy5.5‐Cy3‐FITC was prepared by substituting HPN with HPN‐Cy5.5‐Cy3 and PA‐CAT with FITC‐labeled PA‐CAT. Finally, fluorescence microscopy images of AN‐Cy5.5‐Cy3‐FITC were obtained using CLSM (HP Apo TIRF 100X N.A. 1.49, Nikon, Ti‐E‐A1R, Japan).

The Vehicle without HPN was prepared as follows. Membrane‐less PDDA/dsDNA coacervate microdroplet dispersions were first formed by mixing 0.5 mL of dsDNA aqueous solution (10 mg mL^−1^) and 0.3 mL of PDDA aqueous solution (10 mg mL^−1^). Subsequently, 0.8 mL of a suspension of PDDA/dsDNA coacervate microdroplets was added to 40 µL of 20 mg mL^−1^ DOPC ethanol solution, achieving a final weight ratio of 10% (DOPC: coacervate, w/w). After aging for over 2 h at room temperature, the suspension (10 mg mL^−1^) was mixed with 50 µL of PA‐CAT solution (5 mg mL^−1^). Following an incubation period of 120 min and subsequent centrifugation for 5 min at 20  ×  g, Vehicle were obtained.

### Cell Culture

HUVECs (ATCC, CRL‐1730) and Raw 264.7 cells (ATCC, TIB‐71) were cultured in high‐glucose DMEM (Gibco) supplemented with 10% foetal bovine serum (Sperikon Life Science & Biotechnology Co., Ltd), 100 U mL^−1^ of penicillin G sodium, and 100 µg mL^−1^ of streptomycin sulfate (Hyclone) in a humidified atmosphere containing 5% CO_2_ at 37 °C. All cell lines were confirmed negative through mycoplasma testing.

### Active Chemotactic Behavior of AN in Microfluidic Channels

A dish measuring 10 ×10 mm with HUVECs was prestimulated for 24 h using LPS (1 µg mL^−1^). A three‐inlet and one‐outlet glass substrate microfluidic channel, with dimensions of 2.2 cm (length) × 1.5 mm (width) × 300 µm (height), was utilized to assess the active chemotactic behavior of the AN under CLSM. AN‐Cy5.5 or GCV‐Cy5.5 suspension was introduced into the middle channel at a flow rate of 0.4 mL h^−1^, while the prestimulated HUVEC lysates were introduced into the lower channel at the same flow rate, and normal HUVEC lysates were introduced into the upper channel. Once the flow rate stabilized, CLSM scanning commenced near the outlet tangent, continuously recording the position for 1 min (30 fps) on video. The fluorescence intensity of each material was measured perpendicular to the flow direction using Image J (Version 2.0.0), and a normalized fluorescence intensity curve was plotted based on the average fluorescence intensity of each frame. For the control, normal HUVEC lysates were injected at the same flow rate into both the upper and lower channels, while AN‐Cy5.5 or GCV‐Cy5.5 suspension was introduced into the middle channel at a flow rate of 0.4 mL h^−1^ to monitor the corresponding index.

### AO7 Bleaching Study

The azo dye AO7 was subjected to overnight incubation with varying concentrations of AN. The relative absorbance of AO7 was quantified at 484 nm using a microplate reader (484 nm; MULTISKAN GO, ThermoFisher Scientific, USA).

### In Vitro Biocompatibility Assessment

HUVECs and Raw 264.7 cells were cultured in 96‐well plates at a density of 1 × 10^4^ cells per well and allowed to adhere. Subsequently, they were exposed to different AN densities (4, 8, 12, 16, 20 × 10^3^ cells mL^−1^). Following a 24 h incubation period, cell viability was assessed using the CCK‐8 assay (Beyotime Biotechnology, China).

### In Vitro Cellular Uptake

HUVECs were cultured in glass‐bottom microscope dishes (35 mm in diameter, NEST) at a density of 1 × 10^5^ cells per well until adherence. Cells were prestimulated with 1 µg mL^−1^ of LPS for 24 h. Post‐stimulation, the culture medium was replaced with fresh DMEM containing LPS (1 µg mL^−1^) and either Cy5.5‐labeled membrane‐less PDDA/HPN/dsDNA coacervate microdroplets, GCV‐Cy5.5, or AN‐Cy5.5 (2×10^4^ cells mL^–1^). After a 24‐h incubation, the cellular uptake of the samples was visualized using CLSM (HP Apo TIRF 100X N.A. 1.49, Nikon, Ti‐E‐A1R, Japan).

### Bacterial Culture

*S. aureus* were cultured in an LB medium (10 mg mL^−1^ tryptone, 5 mg mL^−1^ yeast extract, and 0.5 mg mL^−1^ NaCl) at 37 °C on a shaker bed at 200 rpm.

### In Vitro Antibacterial Activity

*S. aureus* were cultured in an LB medium (10 mg mL^−1^ tryptone, 5 mg mL^−1^ yeast extract, and 0.5 mg mL^−1^ NaCl) at 37 °C on a shaker bed at 200 rpm. The resulting bacterial solution incubated at 37 °C for 12 h and was diluted to 1 × 10^6^ CFU mL^−1^ with broth for subsequent experiments. Each well of a 96‐well plate received 20 µL of the bacterial suspension (1×10^6^ CFU mL^−1^) and 160 µL of broth. PBS and varying densities (2.5–20 × 10^3^ cells mL^−1^) of AN, GCV, and Vehicle were added to separate wells. The plate was then incubated at 37 °C on a shaker bed at 200 rpm for 8 h. After incubation, absorbance at 600 nm was measured using a microplate reader (MULTISKAN GO, ThermoFisher Scientific, USA). Each sample was tested in triplicate.

For LB‐agar plate analysis, AN, GCV, and Vehicle suspensions (2 × 10^4^ cells mL^−1^) were added to separate *S. aureus* suspensions (1 × 10^6^ CFU mL^−1^). After a 2‐h incubation, 100 µL of the diluted bacterial suspensions (1:10 000 dilution) was plated onto agar plates. The plates were then incubated at 37 °C for 24 h, and digital photos of the plates were captured for analysis.

### Prosthetic VGI Model

The study adhered to the guidelines outlined in the Guide for Care and Use of Laboratory Animals by the National Institutes of Health. Ethical approval was obtained from the Ethics Committee of Drum Tower Hospital, Medical School of Nanjing University, China, and all animal care and procedures were conducted accordingly. Following a protocol adapted from previous literature,^[^ ^34^ ^]^ a mouse model of prosthetic VGI was established. Briefly, female BALB/c mice aged 6–8 weeks and weighing 18–20 g were anesthetized using 1–5% isoflurane mixed with oxygen via inhalation. The back hair was shaved, and the skin was cleaned with a 10% povidone‐iodine solution. A subcutaneous pocket was created on the right side of the median line through a 0.8 cm incision. A sterile expanded polytetrafluoroethylene graft (0.25 cm^2^, TERUMO, China) was aseptically implanted into the pocket. The incision was closed with 6‐0 polypropylene sutures, and sterile PBS (100 µL) containing *S. aureus* at a concentration of 2×10^7^ CFU mL^−1^ was inoculated onto the graft surface using a tuberculin syringe to form a subcutaneous fluid‐filled pouch. All procedures were performed under sterile conditions, and the animals were monitored daily post‐operation.

### In Vivo Active Chemotactic Effects and Biodistribution of AN

AN‐Cy5.5 and GCV‐Cy5.5 were administered via tail vein injection (8×10^5^ cells mL^−1^, 200 µL) 24 h post‐modeling. As a blank control, 200 µL of PBS was injected into the tail vein. The GCV‐Cy5.5 and PBS groups each consisted of three mice, while the AN‐Cy5.5 group included nine mice. At 6, 12, or 24 h post‐injection, the mice were anesthetized using 1–5% isoflurane mixed with oxygen, and fluorescent images were captured at Ex: 680 nm, Em: 720 nm using a fluorescence imaging system (IVIS Spectrum, PerkinElmer). Subsequently, the animals were euthanized, and skin tissue at the prosthetic vascular graft site along with major organs were harvested and reimaged. The acquired images were analyzed using IVIS Spectrum, living image system 4.5.5 software.

Following imaging, the collected skin tissue at the prosthetic vascular graft site and major organs were rinsed with PBS, blotted with filter paper, and weighed. The tissues were then homogenized in grinding tubes with RIPA lysate (1 mL:100 mg tissue ratio) using a tissue grinder. After centrifugation at 810  ×  g for 5 min, the supernatant was collected and adjusted to 2 mL with RIPA lysate. The fluorescence intensity of the material was measured using a microplate reader (Spark, TECAN, Switzerland; Ex: 680 nm, Em: 720 nm). The amount of AN in each tissue was determined based on the fluorescence intensity‐density standard curve.

### In Vivo Antibacterial Efficiency

The model mice were randomly divided into five groups. AN, GCV, and Vehicle were injected via the tail vein (8 × 10^5^ cells mL^−1^, 200 µL) in three groups, while PBS (200 µL) was injected as a control. The fifth group received a subcutaneous injection of 50 µL of AN. Treatments were administered on days 1, 3, 5, and 7 post‐modeling. On day 8, all mice were euthanized, and the grafts were explanted under sterile conditions for bacteriological examinations. The perigraft tissue was debrided and fixed in a 10% formaldehyde solution for histological analysis using HE, Masson, and CD31 staining. Optical microscope images were taken for HE and Masson staining, while CD31 immunofluorescence images were captured using a CLSM.

The explanted grafts were washed in sterile saline solution, sonicated in phosphate‐buffered saline solution for 5 min to remove adherent bacteria, and quantification of viable bacteria was conducted by preparing serial ten‐fold dilutions (0.1 mL) of bacterial suspensions in 10 mm buffer. Each dilution was cultured on LB agar plates and incubated at 37 °C for 24 h to evaluate the presence of *S. aureus*. Digital photos of the plates were taken, and the number of colonies per plate was counted.

### In Vivo Biosafety Assessment

BALB/c mice were intravenously injected with 200 µL of AN (8×10^5^ or 2×10^5^ cells mL^−1^), with PBS injected into tail vein as the control (200 µL). After 24 h, the mice were euthanized, and blood and major organs were harvested. A routine blood test instrument was used for blood routine examination (WBC, RBC, PLT, HGB) using whole blood, while sections of major organs were stained with HE to evaluate histological changes.

### Antibacterial Efficiency of AN in a Rat Abdominal Aortic VGI Model

Female SD rats weighing 300–350 g were anesthetized using 1–5% isoflurane mixed with oxygen via inhalation. The retroperitoneal space was approached transperitoneally through a midabdominal incision. After the aorta was crossclamped above and below the patch‐suturing region, a 1.5 × 1.0 mm expanded polytetrafluoroethylene graft was sutured on the anterior region of the aortic wall by using 8‐0 polypropylene sutures. Thereafter, *S. aureus* (2 × 10^7^ CFU mL^−1^) was inoculated onto the graft surface. The volume of bacterial suspension was set to 0.1 mL. The animals were divided into three groups: a control group wherein 1 mL of PBS was injected into the tail vein after 5 min of *S. aureus* inoculation; an AN group wherein AN (8×10^5^ cells mL^−1^, 1 mL) was injected into the tail vein after 5 min of *S. aureus* inoculation; and an AN‐L group wherein AN (8×10^5^ cells mL^−1^, 1 mL) was administrated onto the graft surface after 5 min of *S. aureus* inoculation. The retroperitoneum was closed with a 6‐0 polypropylene suture. Heparin was not administered during the operation, and systemic antibiotics were not administered during the operation or in the perioperative period.

On day 4, all mice were euthanized, and macroscopic pictures of the vascular graft and its surrounding tissues were taken to determine the severity of the infection. Then the grafts were explanted under sterile conditions for bacteriological examinations. The explanted grafts were washed in sterile saline solution, sonicated in phosphate‐buffered saline solution for 5 min to remove adherent bacteria, and quantification of viable bacteria was conducted by preparing serial ten‐fold dilutions (0.1 mL) of bacterial suspensions in 10 mm buffer. Each dilution was cultured on LB agar plates and incubated at 37 °C for 24 h to evaluate the presence of S. aureus. Digital photos of the plates were taken, and the number of colonies per plate was counted.

### Statistical Analysis

Statistical analysis was performed using a one‐way analysis of variance (ANOVA) with the Tukey post hoc test in GraphPad Prism software (version 8.4.0). Statistical significance is denoted by asterisks (^*^ *p* <0.05, ^**^ *p* <0.01, ^***^ *p* <0.001).

## Conflict of Interest

The authors declare no conflict of interest.

## Author Contributions

W.J. and H.X. contributed equally to this work. C.M. and M.Z. performed conceptualization. W.J. and M.W. performed methodology. W.J., H.X., Z.W., Z.Z., Y.W., and H.K. performed investigation. W.J., Z.G., and L.W. performed visualization. C.M., M.W., and M.Z. performed supervision. W.J., H.X., and L.W. wrote the original draft. C.M., M.W., and M.Z. reviewed and edited the final manuscript.

## Supporting information

Supporting Information

Supplemental Movie 1

Supplemental Movie 2

## Data Availability

The data that support the findings of this study are available from the corresponding author upon reasonable request.
