# Supplementary material for: Artificial Neutrophils Against Vascular Graft Infection
Source: Adv Sci (Weinh). 2024 Jun 14;11(30):2402768. doi: 10.1002/advs.202402768 (PMC11321623; doi:10.1002/advs.202402768)
Supplement: Supplementary file 1 — Supporting Information [file ADVS-11-2402768-s001.docx]

Supporting Information

**Artificial Neutrophils Against Vascular Graft Infection**

*Wentao Jiang, Huizi Xu, Zheng Gao, Ziyu Wu, Zichun Zhao, Jun Wang, Yawen Wu, Haifeng Ke , Chun Mao*, Mimi Wan**, Min Zhou****

*Corresponding author. Email: maochun@njnu.edu.cn (C.M.); wanmimi@njnu.edu.cn (M.W.); zhouminnju@nju.edu.cn (M.Z.)

**This file includes:**

Figures S1 to S10

Table S1

**Other Supporting Information for this manuscript include the following:**

Movies S1 to S2


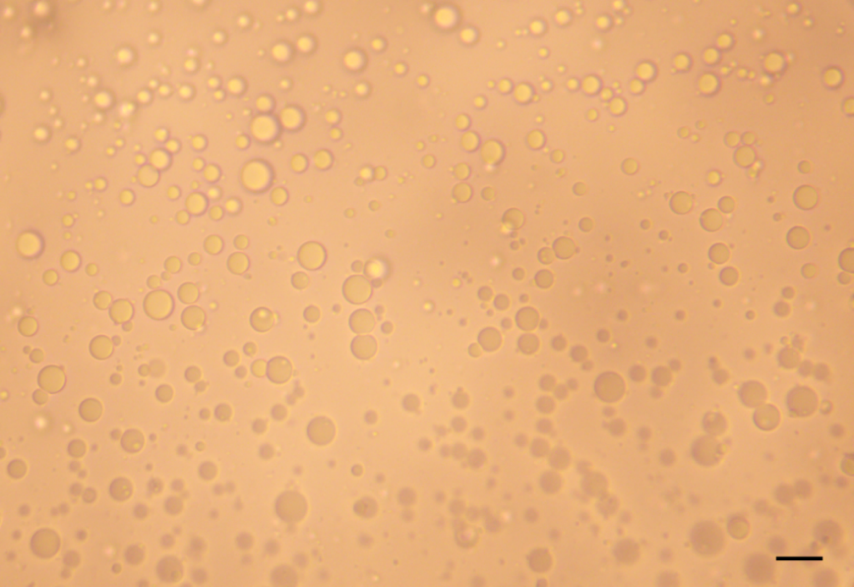


**Figure S1**. Brightfield microscopy image of PDDA/HPN/DNA membrane-free coacervate droplets (scale bar, 10 μm).

**Figure S2.** Zeta potential graph of AN in aqueous solution.


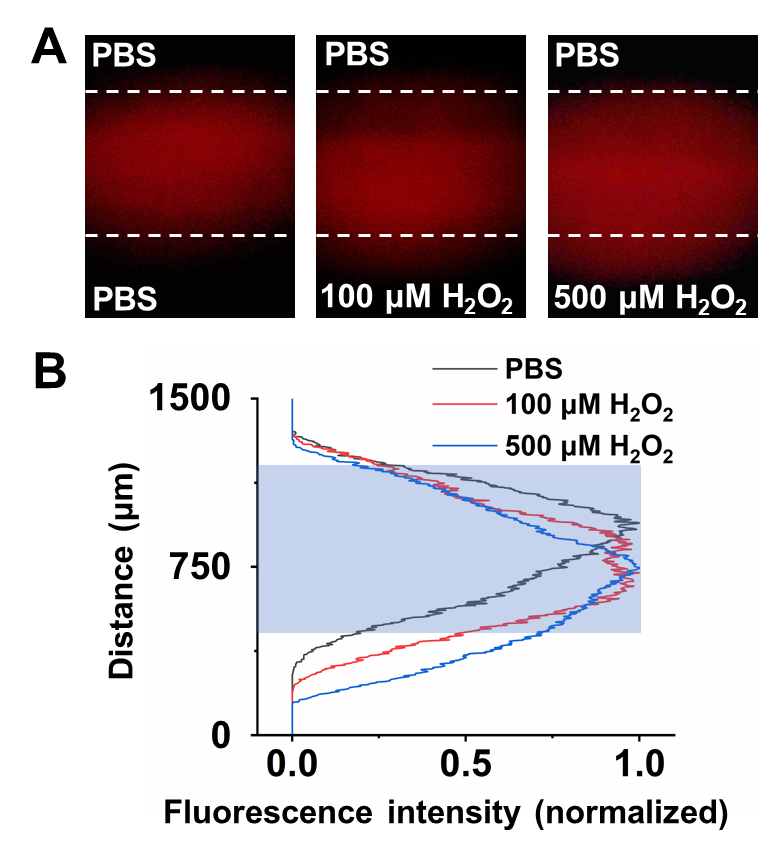


**Figure S3.** (A) Representative CLSM images depicting microfluidic channels for AN traversing the middle channel, with PBS buffer passing through the upper channel, and H_2_O_2_ solutions of different concentrations passing through the lower channel (n = 3 independent experiments). A typical volume flow rate of 0.4 mL h^-1^ through each inlet was maintained to ensure interaction time. (B) Graphs displaying fluorescence intensity distribution normalized to a value between 0–1.


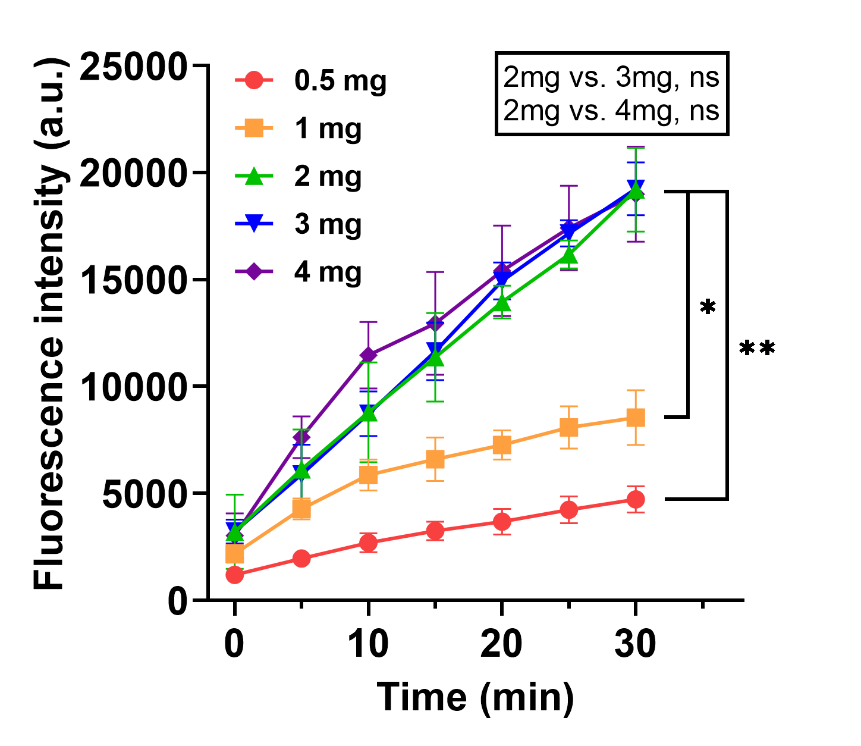


**Figure S4.** Fluorescence changes observed ZIF-8 nanoparticles with different GOx/CPO ratios in PBS solutions with glucose during a 30-min incubation.


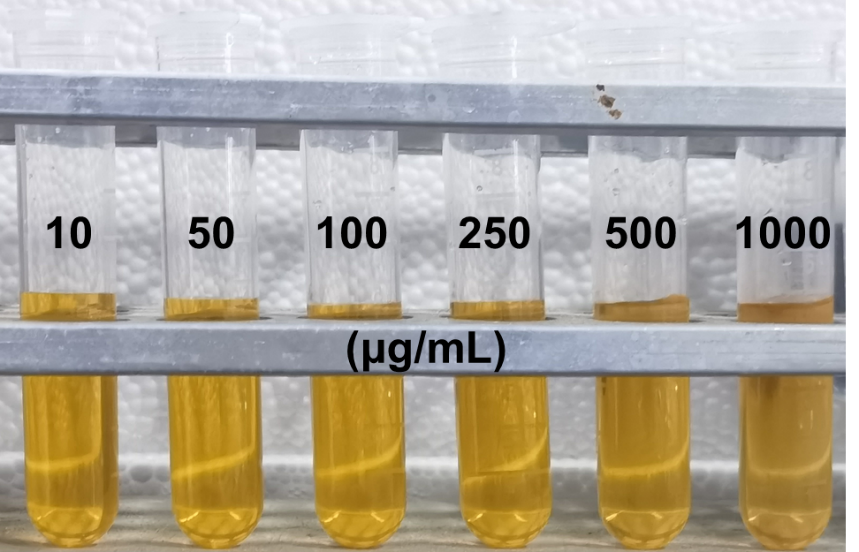


**Figure S5.** The photos of AO7 decolorization using different concentrations of HPN.


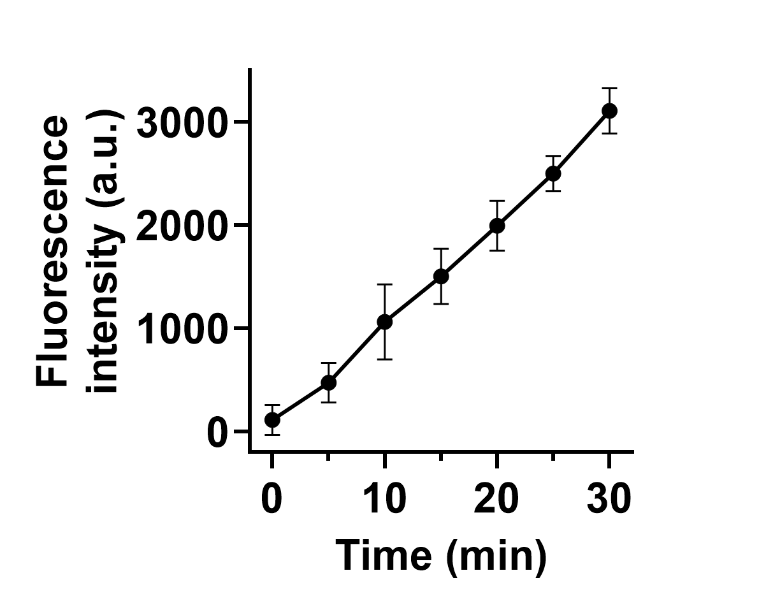


Figure S6. Fluorescence changes observed in AN in culture medium conditions during a 30-min incubation.


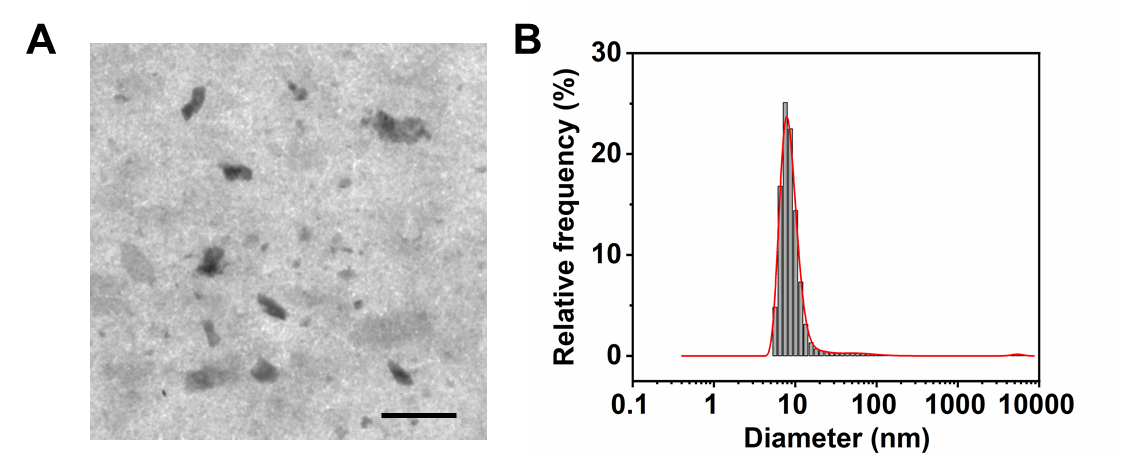


**Figure S7.** (A) TEM image of AN in 10% FBS solution after 48 h (scale bar, 500 nm). (B) The DLS measurement graph of AN in 10% FBS solution after 48 h.


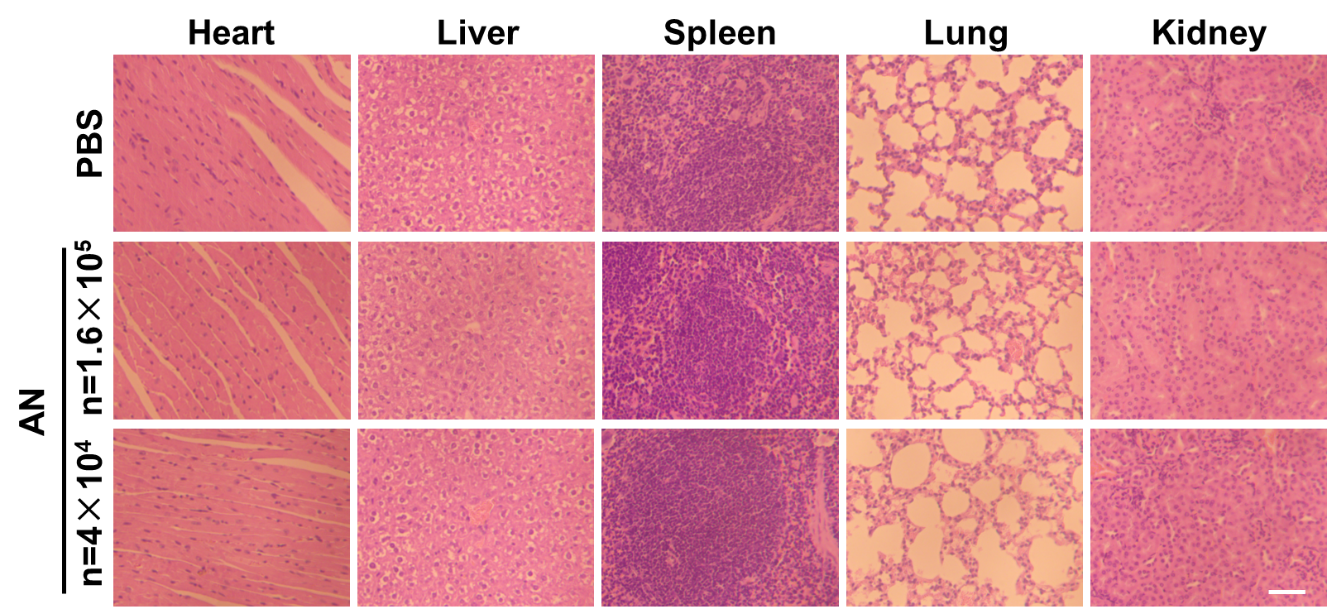


**Figure S8.** HE staining of main organ slices in normal mice after 24 hours of tail vein injection of different numbers of AN and PBS (scale bar, 50 μm).


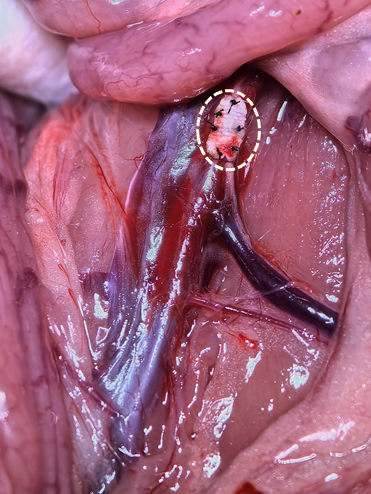


**Figure S9.** Successful establishment of the VGI model in rat abdominal aorta.


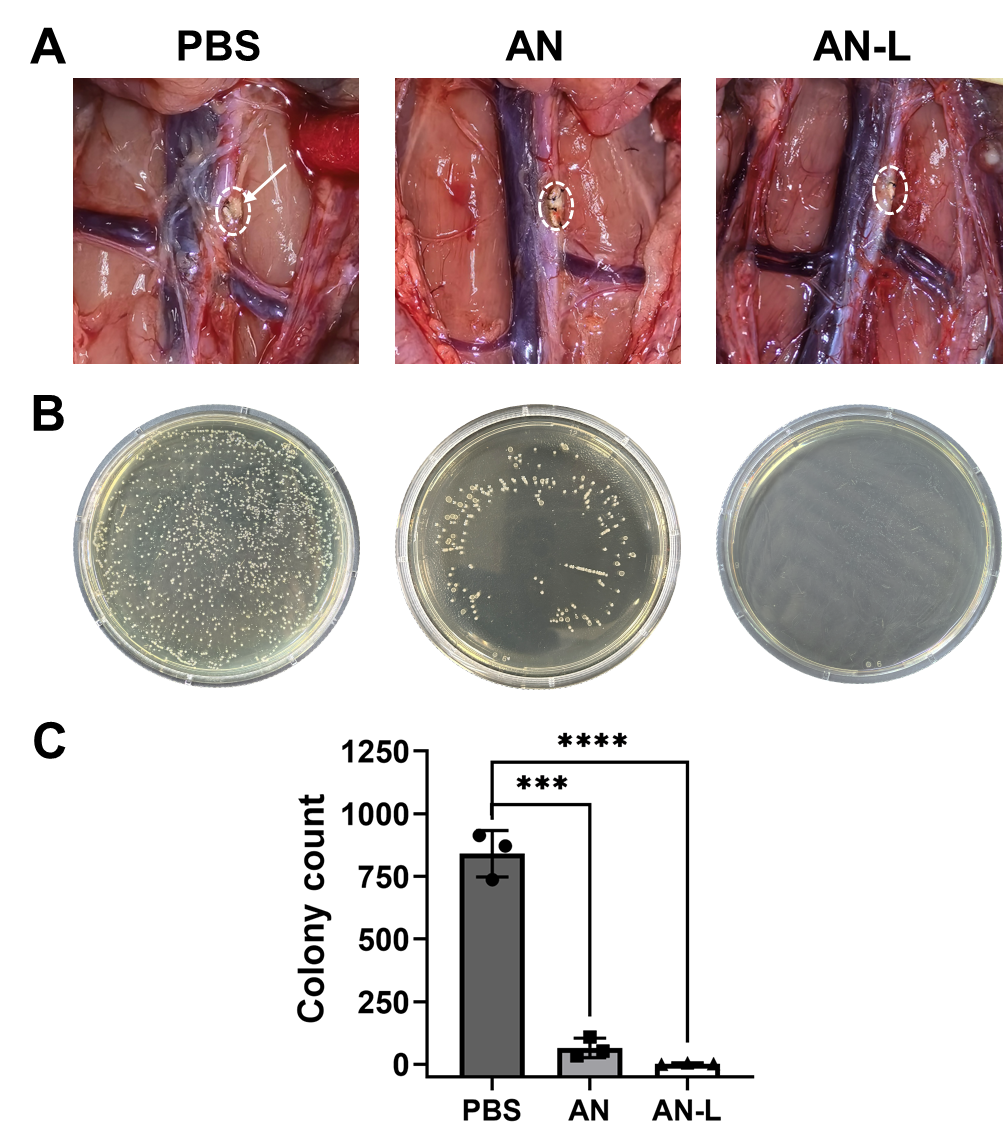


**Figure S10.** (A) Macroscopic findings of the surgical site on day 4. PBS group (control): periprosthetic abscess and purulent fluid accumulation were observed. AN and AN-L group: no infection was observed. (B) Representative image of *S. aureus* colonies on LB agar plates of rat abdominal aorta VGI models treated with different materials on day 4, with (C) corresponding statistical analysis.

**Table S1.** Blood routine results of normal mice after 24 hours of tail vein injection of 1.6×10^5^ artificial neutrophils or 200 μl PBS per mouse.

| Makers | PBS | | | AN | | | |
| --- | --- | --- | --- | --- | --- | --- | --- |
| WBC [×10^9^/L] | 4.3 | 2.97 | 9.17 | | 9.82 | 8.03 | 5.01 |
| RBC [10^12^/L] | 8.59 | 9.19 | 9.3 | | 9.57 | 9.86 | 9.15 |
| PLT [10^9^/L] | 270 | 136 | 724 | | 813 | 676 | 532 |
| NEUT [10^9^/L] | 0.26 | 0.05 | 0.1 | | 0.25 | 0.12 | 0.07 |
| LYMPH [10^9^/L] | 3.41 | 2.19 | 8.18 | | 8.27 | 7.3 | 4.08 |
| MONO [10^9^/L] | 0.04 | 0.01 | 0.03 | | 0.03 | 0.03 | 0.01 |
| BASO [10^9^/L] | 0.57 | 0.71 | 0.86 | | 1.27 | 0.58 | 0.85 |
| NEUT [%] | 6 | 1.8 | 1.1 | | 2.6 | 1.5 | 1.4 |
| LYMPH [%] | 79.3 | 73.7 | 89.2 | | 84.2 | 90.9 | 81.4 |
| MONO [%] | 0.9 | 0.3 | 0.3 | | 0.3 | 0.4 | 0.2 |
| BASO [%] | 13.3 | 23.9 | 9.4 | | 12.9 | 7.2 | 17 |
| HGB [g/L] | 132 | 136 | 146 | | 145 | 151 | 136 |
| HCT [%] | 42.7 | 45.1 | 49.7 | | 48.8 | 50.7 | 45.1 |
| MCV [fL] | 49.7 | 49.1 | 53.4 | | 51 | 51.4 | 49.3 |
| MCH [pg] | 15.4 | 14.8 | 15.7 | | 15.2 | 15.3 | 14.9 |
| MCHC [g/L] | 309 | 302 | 294 | | 297 | 298 | 302 |
| RDW-SD [fL] | 25.2 | 23.6 | 30.4 | | 30 | 30.6 | 27.4 |
| RDW-CV [%] | 19.1 | 18.4 | 20.5 | | 21.1 | 21.2 | 20.3 |

Movie S1. The movie of AN or GCV passing through the middle channel and the normal/prestimulated HUVEC lysate pass through the channels on each side

Movie S2. Laser confocal video of AN and S. aureus co-incubation within 10 min (Green color: S. aureus stained with FTIC, Red color: AN stained with Cy5.5).
